# Arguments about face masks and Covid-19 reflect broader methodologic debates within medical science

**DOI:** 10.1007/s10654-021-00735-7

**Published:** 2021-03-16

**Authors:** Neil Pearce, Jan Paul Vandenbroucke

**Affiliations:** 1 Department of Medical Statistics, London School of Hygiene and Tropical Medicine, London, UK; 2 Department of Clinical Epidemiology, Leiden University Medical Center, Leiden, The Netherlands; 3 Department of Clinical Epidemiology, Aarhus University, Aarhus, Denmark

**Keywords:** Epidemiology, Methods, Evidence synthesis, Causal inference, Causality

## Abstract

There has perhaps been no issue as contentious in Covid-19 as face masks. The most contentious scientific debate has been between those who argue that “there is no scientific evidence”, by which they mean that there are no randomized controlled trials (RCTs), versus those who argue that when the evidence is considered together, “the science supports that face coverings save lives”. It used to be a ‘given’ that to decide whether a particular factor, either exogenous or endogenous, can cause a particular disease, and in what order of magnitude, one should consider all reasonably cogent evidence. This approach is being increasingly challenged, both scientifically and politically. The scientific challenge has come from methodologic views that focus on the randomized controlled trial (RCT) as the scientific gold standard, with priority being given, either to evidence from RCTs or to observational studies which closely mimic RCTs. The political challenge has come from various interests calling for the exclusion of epidemiological evidence from consideration by regulatory and advisory committees.

## Introduction

There has perhaps been no issue as contentious in Covid-19 as face masks. Their benefits and drawbacks are endlessly debated politically, and scientifically. The most contentious scientific debate has been between those who argue that “there is no scientific evidence”, by which they actually mean that there are no randomized controlled trails (RCTs), vs. those who argue that, admittedly, there is no single definitive study, but when the evidence is considered together, “the science supports that face coverings save lives” [1]. Advocates of the latter approach would argue that there is also no RCT evidence that parachutes save lives [2], but that there is a strong hunch based on knowledge of humans falling from altitudes.

It used to be a ‘given’ that to decide whether a particular factor, either exogenous or endogenous, can cause a particular disease, and in what order of magnitude, one should consider all reasonably cogent evidence: several types of epidemiologic studies, clinical studies, laboratory studies, and sometimes socio-economic studies. This approach is being increasingly challenged. The scientific challenge has come from methodologic views that focus on the randomized controlled trial (RCT) as the scientific gold standard. However, the form of the argument has changed over time. In particular, we can distinguish between an earlier phase when the main distinction (as drawn, for example, by the Cochrane Collaboration [3], and Evidence-Based Medicine [4]) was between RCTs and observational studies, and more recent developments where a distinction has been drawn between observational studies conducted using an RCT paradigm with the intention of mimicking a ‘target trial’ (e.g. causal inference methods [5]), and other observational studies [6].

The political challenge has come from various interests, skilfully employing calls for ‘greater accountability in science’ [7]. This has been particularly motivated by anti-regulation “vested interests” which have disputed a long series of scientific discoveries, ranging from smoking and lung cancer to the reality of climate change [8–10]. Recently, these pressures have led to calls for the exclusion of epidemiological evidence from consideration by regulatory and advisory committees, thereby weakening regulatory standards [11]. For example, a recent appointee to the EPA Clean Air Scientific Advisory Committee argued that *“rather than relying on the weight-of-evidence approach that the EPA has traditionally used to infer causation*, [the Clean Air Science Advisory Committee] *wants to rely on studies that use… ‘manipulative causality’”.* This restricts acceptable evidence to assess causality to results from RCTs or observational studies on interventions that were analysed with the use of causal inference statistical methods [11]. Similar views have been expressed at high levels of the current British Government [12].

## Evidence synthesis

Evidence synthesis may include systematic reviews or formal meta-analyses of the findings of observational studies and/or RCTs, but it usually also incorporates other types of evidence, e.g. animal studies and laboratory science. Several key considerations for adjudicating causality were proposed by Bradford Hill in 1965 [13]: Strength; Consistency; Specificity; Temporality; Biological gradient; Plausibility; Coherence; Experiment; and Analogy. Bradford Hill stresses that these are *considerations* rather than criteria, and that no single consideration is essential for causality to be inferred, but they do provide a framework for the synthesis of available evidence. Since 1972, the International Agency for Research on Cancer (IARC) Monographs provide a model for a systematic approach to combine evidence from human, animal and mechanistic studies to classify various exposures with regards to carcinogenicity.

In such approaches, inferring causality involves the synthesis of a variety of evidence, and no single study is definitive [13], not even a randomized trial. In fact, many important questions cannot be studied by RCTs (think of environmental, occupational effects, but also long-term adverse effects of medical therapies), not all RCTs are done well, and the interpretation of whether they are also involves judgment.

An integrative view of evidence synthesis that has come under renewed attention recently is named ‘triangulation’ [14]. It explores various potential biases by comparing studies and populations where the hypothetical biases are likely to be in different directions. If the studies tend to be concordant regarding the observed association, especially if their different potential biases are in opposite directions, this supports a causal interpretation. Importantly, this is in contrast to standard meta-analysis which aims at finding and combining similar studies to reach one overall estimate, Triangulation focusses on identifying different study types and different populations in which one would expect the biases to be *different and in different directions*, so that the likely strength of the hypothesized biases can be assessed.

Triangulation strategies include cross-context comparisons, use of different control groups, natural experiments, within-sibling comparisons, natural experiments, instrumental variable (IV) analyses, Mendelian Randomization (MR), exposure-negative controls, and outcome-negative controls. Other non-RCT-based methods include population comparisons, difference in difference, and regression discontinuity studies [15]. Of course, some specific studies may carry greater weight than others, but this is highly topic and context-specific.

Thus, traditional approaches to evidence synthesis, including triangulation of evidence, are pluralistic and inclusive, with all of the relevant evidence being considered. For example, in the IARC Monographs on carcinogenicity, laboratory and animal studies are playing an increasingly important role, next to human data [16].

## The challenge arising from translating theories about the superiority of RCTs and ‘causal inference’, into scoring systems

Both phases in the debates about the RCT being the gold standard, have led to the development of scoring systems for evidence.

The older view from the Evidence-Based Medicine movement prioritized RCTs for medical decision making, in an iconic ‘pyramid of evidence’ with the RCT on top, followed at some large distance by observational cohort studies and at even further distance for other types of observational studies. This thinking was crystallised in scoring systems like GRADE that in practice are completely geared towards RCTs; they will only ‘upgrade’ observational studies if there is evidence of ‘reverse confounding’, or if the effect is huge [17]. This pyramid may be useful for certain medical decisions, e.g., about drugs with small benefits. However, it has been argued repeatedly how this it fails for other types of medical and public health knowledge [18–20].

One recent controversy [21] involved an analysis of studies of processed and red meat. This used the GRADE criteria to exclude virtually all of the observational study evidence, leaving only two intervention studies; however, these intervention studies were of rather debatable relevance because of their short follow-up and uncertainty of the difference between people with different dietary habits [22–24]. In contrast, the Working Group of the IARC Monographs considered all of the available evidence, and concluded that the evidence for an increased risk of colorectal cancer was convincing for processed meat and probable also for red meat [25]. In the integrative approach to evidence synthesis, the maxim is that all studies have potential limitations, but that one should carefully look and judge each limitation and whether it can be overcome by other studies or other types of evidence.

In recent years a ‘causal inference’ theory has increasingly promoted a distinction within observational studies, based on the idea an RCT is the gold standard for research involving exposures and outcomes in humans. Thus, when we cannot perform an RCT, the next best option is to perform an observational study which closely mimics the RCT model [26]. This has led to new scoring systems such as ROBINS-I, ROBINS-E [27, 28]. We have argued elsewhere [29], that this ‘mimic the RCT approach’ limits epidemiologic research to ‘events’ or interventions and ignores other types of evidence, e.g. time trends or ecological comparisons may make an important contribution to evidence synthesis. More specifically, a scoring system demanding an RCT-like framework for observational studies also downgrades events/interventions that are not followed from initiation of exposure onwards. Elsewhere, it has been argued why the latter would a priori downgrade long-term epidemiologic studies on smoking, occupational and environmental health [6, 30]. Moreover, causal inference theory not only excludes (or strongly downgrades) much useful evidence; it is also ‘politically conservative’ as it cannot envisage interventions on societies to reduce social inequalities, or on the planet, as are needed for climate change [31].

Nevertheless, the RCT-paradigm has become increasingly dominant in epidemiological theory in recent years [29]. The zenith (or nadir, depending on your point of view) of this approach, has been the development of scoring systems which are used to assess individual studies, and score them according to whether they are RCTs, or score them relative to the ‘gold standard’ of a hypothetical RCT. Such scoring systems result in many thoughtfully executed observational studies being scored as ‘low quality’, and being effectively excluded from consideration, even though they may be very illuminating when considered together with other studies. The present Covid-19 epidemic is a case in point: all actions to mitigate the spread of the epidemic are based on (admittedly partial) understanding of this particular type of virus transmission, insights about the viral genome, modelling of observational data of counts and rates, and comparisons between actions of regions or countries.

## The challenge from vested interests

Traditional approaches to evidence synthesis, much like any science, from astronomy to physics, inevitably involve subjective judgements at the forefront of scientific developments, e.g. when comparing the findings of studies which used different designs, which were conducted in different populations, or when comparing and synthesizing epidemiological, clinical, animal, and mechanistic evidence. It should be emphasized that equally subjective judgments can play a role in accepting or not accepting the results of RCTs—think of all debates about the validity of RCTs organized by the pharmaceutical industry which makes the medications that they put themselves on trial.

Integrative considerations such as exemplified by those of Bradford Hill, as well as those used on a regular basis nowadays by IARC, provide a powerful guide of how to synthesize different types of evidence. Of course, decision-making processes may vary depending on the urgency of the issue (e.g. Covid-19), and the consequences to individuals and to the public health of an incorrect decision [32]. Nevertheless, these considerations play their part in a context where it is increasingly common for decisions of regulatory committees to be challenged, or for different committees to reach different conclusions on the basis of the same set of evidence. This has left regulatory bodies open to legal challenge, which have been increased by recent political developments that resulted in greater representation of vested interests on regulatory matters [7]. In turn, such regulatory bodies have an understandable desire for a more transparent decision-making process, to protect themselves from litigation. Thus, having a clear set of rules (no matter how simplistic), may be seen as more convenient politically and legally, in comparison with the standard scientific decision-making process which inevitably involves judgements [33].

To placate legal and vested interests, it looks as if an easy solution is at hand, in that there is now a ready-made methodological view which explicitly argues that RCT-type evidence is the gold standard, and other observational evidence is of doubtful, if any, validity. Moreover, there are now readily available tools (GRADE, ROBINS-I, etc.) which can be used to score individual studies on humans, clinical as well as epidemiological, on this basis. Such scoring systems have a veneer of objectivity, or at least provide a clear ‘paper trail’ so that regulatory authorities can more transparently report how decisions were made.

The quote from the recent appointee to the EPA Clean Air Scientific Advisory Committee that we mentioned speaks for itself. It prefers a theory that restricts numerical evidence to intervention studies or analyses with the use of causal inference statistical methods [11].

We want to emphasize that, despite the disturbing aspects of these developments, we are not arguing that there is any direct connection between modern causal inference theory and attempts to influence evidence synthesis by vested interests. However, both reinforce each other’s tendency to restrict the ‘acceptable evidence’ to studies which are RCTs, or observational studies which closely mimic RCTs. All other relevant evidence from individual studies (triangulation, time trends, animal studies, mechanistic studies) does not fit this paradigm, and such studies are either rejected or scored so low that they are ignored [34].

## What is to be done?

Covid-19 has, within nine months, provided a ‘fast forward’ version of ‘normal science’ in which decisions need to be made quickly, lives are at stake, and one cannot wait for the perfect study. This does not mean that standard RCTs are not important—on the contrary, the trials of dexamethasone, chloroquine, and vaccines illustrate their crucial role, but it means that there are many important issues about curtailing the epidemic for which trials are neither possible, nor required. One example is the acceptance of the cumulative effect of barrier methods and screening to break the chain of transmission, whether they are mouth masks or rapid tests—based on insight and modelling, rather than RCTs or RCT-like observational studies [1].

The way ahead is advocated by Savitz et al. [35] who argue that risk of bias assessments should focus on identifying a (small) number of the most likely influential sources of bias, classifying each study on how effectively it has addressed each potential bias, and then determining whether results differ across studies in relation to susceptibility to each hypothesized source of bias. For example, if non-differential misclassification of exposure (which usually produces a bias towards the null) is likely to be a problem in some studies, but if these studies all yield similar positive findings, and other studies with likely less misclassification of exposure yield even stronger positive findings, then misclassification of exposure is unlikely to explain the findings of the former studies.

Algorithm-based methods such as GRADE and ROBINS-I that rely on the RCT paradigm are just a part of the toolkit of methods that can be used for evidence synthesis. When used carefully, they may assist the assessment of possible biases in studies of some particular exposure-outcome associations. For example, being able to group studies as to the likely occurrence, direction and strength of residual confounding, may provide a useful knowledge base for triangulation, and for the approach advocated by Savitz et al. [35]. However, when these scores are used inappropriately to score individual studies, and to reject standard observational evidence on the basis of such scores, these algorithm-based systems have considerable potential for harm, both to science and to the health of the public.

## Data Availability

Not applicable.
